# *catena*-Poly[[[aqua­(propane-1,3-diamine-κ^2^ *N*,*N*′)copper(II)]-μ-fumarato-κ^2^ *O*:*O*′] monohydrate]

**DOI:** 10.1107/S1600536808000160

**Published:** 2008-01-09

**Authors:** M. Padmanabhan, James C. Joseph, Susanne Olsson, Mohammed Bakir

**Affiliations:** a School of Chemical Sciences, Mahatma Gandhi University, Kottayam 686 560, Kerala, India; b Department of Chemistry, Göteborg University, SE-41296 Göteborg, Sweden; c Department of Chemistry, The University of the West Indies – Mona Campus, Kingston 7, Jamaica

## Abstract

The asymmetric unit of the title compound, {[Cu(C_4_H_2_O_4_)(C_3_H_10_N_2_)(H_2_O)]·H_2_O}_*n*_, consists of two Cu^II^ atoms, half each of two propane-1,3-diamine ligands and two coordinated water mol­ecules, all lying on crystallographic mirror planes, also one fumarate dianion and one uncoordinated water mol­ecule in a general position. The Cu(C_3_H_10_N_2_)(H_2_O) units are linked *via* fumarate dianions into a zigzag chain running along the *a* axis. A longer Cu—O distance [2.873 (3) Å] is to a water mol­ecule bridging equivalent Cu^II^ atoms in adjacent chains, forming a three-dimensional framework. One of the Cu^II^ atoms is in a distorted square-pyramidal environment and the other is in a pseudo-octa­hedral geometry of the [5+1] type. O—H⋯O and N—H⋯O hydrogen bonds are observed in the crystal structure.

## Related literature

For related literature, see: Chan (2007 ▶); Dong *et al.* (2006 ▶); Mori *et al.* (2005 ▶); Mukherjee *et al.* (2004 ▶); Rudkevich (2007 ▶); Shi *et al.* (2007 ▶); Ye *et al.* (2005 ▶); Zheng & Xie (2004 ▶).
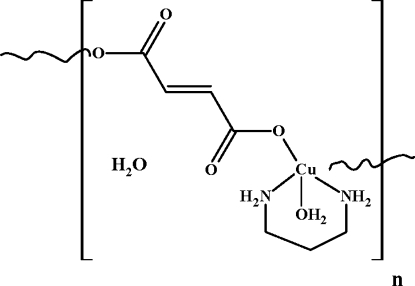


## Experimental

### 

#### Crystal data

[Cu(C_4_H_2_O_4_)(C_3_H_10_N_2_)(H_2_O)]·H_2_O
*M* *_r_* = 286.76
Orthorhombic, 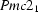

*a* = 14.993 (3) Å
*b* = 8.0948 (17) Å
*c* = 9.259 (2) Å
*V* = 1123.7 (4) Å^3^
*Z* = 4
Mo *K*α radiation
μ = 1.96 mm^−1^
*T* = 293 (2) K
0.30 × 0.20 × 0.10 mm

#### Data collection

Rigaku R-AXIS IIC image-plate system diffractometer
Absorption correction: multi-scan (*CrystalClear*; Rigaku, 2000 ▶) *T*_min_ = 0.543, *T*_max_ = 0.82
10226 measured reflections
3345 independent reflections
2913 reflections with *I* > 2σ(*I*)
*R*_int_ = 0.054

#### Refinement

*R*[*F* ^2^ > 2σ(*F* ^2^)] = 0.041
*wR*(*F* ^2^) = 0.096
*S* = 1.14
3345 reflections
154 parameters
1 restraint
H-atom parameters constrained
Δρ_max_ = 0.47 e Å^−3^
Δρ_min_ = −0.82 e Å^−3^
Absolute structure: Flack (1983 ▶), with 1415 Friedel pairs
Flack parameter: 0.011 (18)

### 

Data collection: *CrystalClear* (Rigaku, 2000 ▶); cell refinement: *CrystalClear*; data reduction: *CrystalClear*; program(s) used to solve structure: *SHELXS97* (Sheldrick, 2008 ▶); program(s) used to refine structure: *SHELXL97* (Sheldrick, 2008 ▶); molecular graphics: *ORTEP-3 for Windows* (Farrugia, 1997 ▶); software used to prepare material for publication: *SHELXL97*.

## Supplementary Material

Crystal structure: contains datablocks I, global. DOI: 10.1107/S1600536808000160/ci2545sup1.cif

Structure factors: contains datablocks I. DOI: 10.1107/S1600536808000160/ci2545Isup2.hkl

Additional supplementary materials:  crystallographic information; 3D view; checkCIF report

## Figures and Tables

**Table:** 

Cu1—N1	2.019 (3)
Cu1—O1	2.024 (2)
Cu1—O3	2.180 (3)
Cu2—N1′	2.010 (3)
Cu2—O1′	2.015 (3)
Cu2—O3′	2.270 (3)

**Table:** 

N1^i^—Cu1—N1	90.42 (18)
N1—Cu1—O1	88.93 (11)
N1—Cu1—O1^i^	173.62 (11)
O1—Cu1—O1^i^	91.00 (14)
N1—Cu1—O3	97.70 (11)
O1—Cu1—O3	88.68 (10)
N1′^ii^—Cu2—N1′	89.83 (19)
N1′—Cu2—O1′	89.47 (10)
N1′—Cu2—O1′^ii^	175.68 (13)
O1′—Cu2—O1′^ii^	90.90 (15)
N1′—Cu2—O3′	95.62 (11)
O1′—Cu2—O3′	88.69 (10)

Symmetry codes: (i) 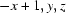; (ii) 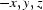.

**Table 2:** Hydrogen-bond geometry (Å, °)

*D*—H⋯*A*	*D*—H	H⋯*A*	*D*⋯*A*	*D*—H⋯*A*
O4—H4*A*⋯O2′	0.87	2.17	2.887 (5)	140
O3—H3⋯O2^iii^	0.85	1.88	2.713 (3)	166
O3′—H3′⋯O2′^iv^	0.85	1.91	2.708 (3)	156
N1—H1*A*⋯O1^v^	0.90	2.20	3.083 (4)	167
N1′—H1′*A*⋯O4^vi^	0.90	2.25	3.071 (5)	151
N1′—H1′*B*⋯O1′^vii^	0.90	2.26	3.135 (3)	164
O4—H4*B*⋯O2^viii^	0.86	1.99	2.785 (4)	153

Symmetry codes: (iii) 
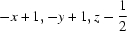
; (iv) 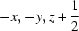; (v) 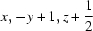; (vi) 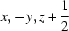; (vii) 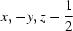; (viii) 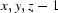.

**Fig. 1.**
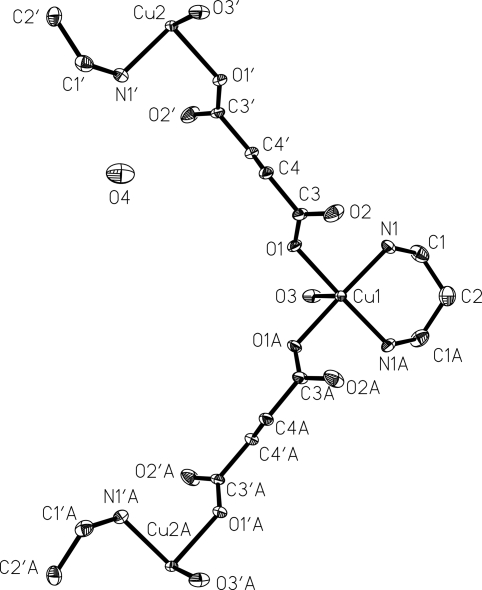
The coordination environment of the CuII center, showing the atom-labeling scheme. Displacement ellipsoids are drawn at the 30% probability level. H atoms have been omitted for clarity. [symmetry code: -x + 1, y, z.]

**Fig. 2.**
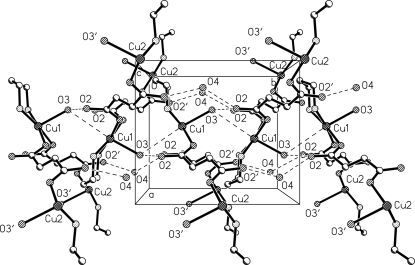
Part of a zigzag polymeric chain of the title compound. Hydrogen bonds are shown as dashed lines.

## supplementary crystallographic information

### Comment

Metallo-polymers are of current interest because of their physical properties and applications in many areas (Chan, 2007; Rudkevich, 2007; Shi *et al.*, 2007; Mori *et al.*, 2005). The synthesis and crystal structure of copper-polycarboxylate polymers have been reported (Mukherjee *et al.*, 2004; Ye *et al.*, 2005). Now we report here the crystal structure of the title compound.

The asymmetric unit of the title compound consists of two Cu^II^ atoms, one half each of two 1,3-diaminopropane ligands and two water molecules, all lying on crystallographic mirror planes, and one fumarate dianion. As shown in Fig.1, the [(aqua)(propane-1,3-diamino-κ^2^-N,*N*)copper(II)] units are linked *via* amphi-monodentate fumarate dianions into a zigzag chain along the *a* axis. Each Cu^II^ atom has a distorted square-pyramidal environment, being coordinated by two N atoms of the 1,3-diaminopropane ligand and two *cis*-oxygen atoms from two bridging fumarate dianions in the basal positions and a water molecule in the apical position. The axial Cu—O bond distance [2.180 (3) Å] is shorter, and the Cu···Cu distance [9.084 Å] is longer than the corresponding distances [2.481 Å and 8.857 Å] reported for fumarate bridged [(aqua)(1,2-dimethylethane-1,2-diamine-κ^2^-N,*N*)copper(II)] (Mukherjee *et al.*, 2004). The six-membered metallocyclic ring formed by the N,*N*-bidentae propane-1,3-diamine ligand and the Cu^II^ atom adopts a chair conformation.

In the crystal structure, the longer Cu2—O3'(-*x*,-*y*,-1/2 + *z*) coordination [2.873 (3) Å] involving the water molecule bridges Cu^II^ atoms of adjacent zigzag chains, leading to the formation a three-dimensional framework. The coordination of the Cu2 atom in the network is pseudo-octahedral of the [5 + 1] type. The structure is further stabilized by O—H···O and N—H···O hydrogen bonds (Table 2). The geometry of hydrogen bonds are similar to those reported for a variety of copper compounds (Zheng & Xie, 2004; Dong *et al.*, 2006).

Due to their convenient synthesis and potential catalytic and sorption applications, studies are in progress in our laboratories to synthesis several other polycarboxylate metallopolymers which are structurally and electronically tuned by polyamines.

### Experimental

Fumaric acid (0.12 g, 1.0 mmol) was added to an aqueous suspension of CuCO_3_.Cu(OH)_2_.H_2_O (0.12 g, 0.50 mmol), and then propane-1,3-diamine (0.08 ml, 1.0 mmol) was added dropwise, with stirring and heating. The mixture was allowed to react for 2 h and filtered, and the filtrate was allowed to stand at room temperature for 4 d. At the end of this time, deep blue colourless crystals deposited, which were filtered off and dried in air.

### Refinement

The water H atoms were located from a difference Fourier map and constrained to ride on their parent atoms with *U*_iso_(H) = 1.5*U*_eq_(O). The remaining H atoms were placed in idealized positions and constrained to ride on their parent atoms, with N—H = 0.90 Å, C—H = 0.93 or 0.97 Å and *U*_iso_(H) = 1.2*U*_eq_(C,*N*).

### Crystal data

**Table:**

[Cu(C_4_H_2_O_4_)(C_3_H_10_N_2_)(H_2_O)]·H_2_O	*F*(000) = 596
*M**_r_* = 286.76	*D*_x_ = 1.701 Mg m^−^^3^
Orthorhombic, *P**m**c*2_1_	Mo *K*α radiation, λ = 0.71073 Å
Hall symbol: P 2c -2	Cell parameters from 6311 reflections
*a* = 14.993 (3) Å	θ = 1.4–25.0°
*b* = 8.0948 (17) Å	µ = 1.96 mm^−^^1^
*c* = 9.259 (2) Å	*T* = 293 K
*V* = 1123.7 (4) Å^3^	Plate, blue
*Z* = 4	0.30 × 0.20 × 0.10 mm

### Data collection

**Table:**

Rigaku R-AXIS IIC image-plate system diffractometer	3345 independent reflections
Radiation source: fine-focus sealed tube	2913 reflections with *I* > 2σ(*I*)
graphite	*R*_int_ = 0.054
Detector resolution: 105 pixels mm^-1^	θ_max_ = 33.0°, θ_min_ = 1.4°
φ scans	*h* = −20→20
Absorption correction: multi-scan (*CrystalClear*; Rigaku, 2000)	*k* = −11→11
*T*_min_ = 0.543, *T*_max_ = 0.82	*l* = −12→11
10226 measured reflections	

### Refinement

**Table:**

Refinement on *F*^2^	Secondary atom site location: difference Fourier map
Least-squares matrix: full	Hydrogen site location: inferred from neighbouring sites
*R*[*F*^2^ > 2σ(*F*^2^)] = 0.041	H-atom parameters constrained
*wR*(*F*^2^) = 0.096	*w* = 1/[σ^2^(*F*_o_^2^) + (0.044*P*)^2^] where *P* = (*F*_o_^2^ + 2*F*_c_^2^)/3
*S* = 1.14	(Δ/σ)_max_ = 0.001
3345 reflections	Δρ_max_ = 0.47 e Å^−^^3^
154 parameters	Δρ_min_ = −0.82 e Å^−^^3^
1 restraint	Absolute structure: Flack (1983), 1415 Friedel pairs
Primary atom site location: structure-invariant direct methods	Flack parameter: 0.011 (18)

### Special details

**Table:**

Geometry. All e.s.d.'s (except the e.s.d. in the dihedral angle between two l.s. planes) are estimated using the full covariance matrix. The cell e.s.d.'s are taken into account individually in the estimation of e.s.d.'s in distances, angles and torsion angles; correlations between e.s.d.'s in cell parameters are only used when they are defined by crystal symmetry. An approximate (isotropic) treatment of cell e.s.d.'s is used for estimating e.s.d.'s involving l.s. planes.
Refinement. Refinement of *F*^2^ against ALL reflections. The weighted *R*-factor *wR* and goodness of fit *S* are based on *F*^2^, conventional *R*-factors *R* are based on *F*, with *F* set to zero for negative *F*^2^. The threshold expression of *F*^2^ > σ(*F*^2^) is used only for calculating *R*-factors(gt) *etc*. and is not relevant to the choice of reflections for refinement. *R*-factors based on *F*^2^ are statistically about twice as large as those based on *F*, and *R*- factors based on ALL data will be even larger.

### Fractional atomic coordinates and isotropic or equivalent isotropic displacement parameters (Å^2^)

**Table:**

	*x*	*y*	*z*	*U*_iso_*/*U*_eq_	
Cu1	0.5000	0.54986 (6)	0.72824 (5)	0.02037 (11)	
Cu2	0.0000	−0.01107 (9)	0.46996 (5)	0.02080 (12)	
O1	0.40374 (16)	0.3988 (3)	0.6507 (2)	0.0289 (5)	
O1'	0.09578 (17)	0.1419 (3)	0.5436 (3)	0.0286 (5)	
O2	0.34823 (19)	0.3174 (4)	0.8619 (3)	0.0505 (8)	
O2'	0.1459 (2)	0.2442 (4)	0.3340 (3)	0.0484 (8)	
O3	0.5000	0.6785 (5)	0.5214 (4)	0.0381 (9)	
H3	0.5511	0.6699	0.4823	0.057*	
O3'	0.0000	−0.1382 (5)	0.6885 (3)	0.0342 (8)	
H3'	−0.0533	−0.1602	0.7130	0.051*	
N1	0.4044 (2)	0.6854 (4)	0.8260 (3)	0.0299 (6)	
H1A	0.3986	0.6477	0.9170	0.036*	
H1B	0.3525	0.6652	0.7804	0.036*	
N1'	0.0947 (2)	−0.1553 (4)	0.3820 (3)	0.0279 (6)	
H1'A	0.1472	−0.1269	0.4218	0.034*	
H1'B	0.0979	−0.1306	0.2874	0.034*	
C1	0.4162 (3)	0.8666 (5)	0.8328 (5)	0.0461 (10)	
H1C	0.3653	0.9154	0.8814	0.055*	
H1D	0.4184	0.9108	0.7355	0.055*	
C1'	0.0848 (3)	−0.3360 (4)	0.3952 (4)	0.0359 (8)	
H1'C	0.0838	−0.3662	0.4965	0.043*	
H1'D	0.1357	−0.3898	0.3507	0.043*	
C2	0.5000	0.9135 (7)	0.9117 (7)	0.0498 (14)	
H2A	0.5000	0.8602	1.0056	0.060*	
H2B	0.5000	1.0320	0.9276	0.060*	
C2'	0.0000	−0.3955 (6)	0.3235 (6)	0.0402 (12)	
H2'A	0.0000	−0.5153	0.3231	0.048*	
H2'B	0.0000	−0.3588	0.2238	0.048*	
C3	0.34705 (18)	0.3246 (4)	0.7272 (4)	0.0272 (5)	
C3'	0.1494 (2)	0.2248 (4)	0.4675 (4)	0.0264 (6)	
C4	0.2697 (2)	0.2419 (4)	0.6537 (3)	0.0275 (6)	
H4	0.2515	0.1388	0.6869	0.033*	
C4'	0.2259 (2)	0.3090 (4)	0.5432 (3)	0.0261 (6)	
H4'	0.2434	0.4129	0.5112	0.031*	
O4	0.2556 (3)	0.1667 (5)	0.0869 (5)	0.0787 (12)	
H4A	0.2359	0.2358	0.1510	0.118*	
H4B	0.2686	0.2327	0.0168	0.118*	

### Atomic displacement parameters (Å^2^)

**Table:**

	*U*^11^	*U*^22^	*U*^33^	*U*^12^	*U*^13^	*U*^23^
Cu1	0.0162 (2)	0.0256 (3)	0.0193 (2)	0.000	0.000	−0.0011 (2)
Cu2	0.0144 (2)	0.0250 (3)	0.0230 (2)	0.000	0.000	−0.00166 (16)
O1	0.0209 (12)	0.0378 (13)	0.0279 (12)	−0.0114 (9)	0.0002 (8)	−0.0035 (9)
O1'	0.0187 (13)	0.0361 (13)	0.0309 (12)	−0.0071 (10)	0.0010 (9)	−0.0060 (10)
O2	0.0413 (17)	0.085 (2)	0.0248 (12)	−0.0221 (15)	−0.0051 (11)	0.0059 (13)
O2'	0.0428 (16)	0.076 (2)	0.0268 (12)	−0.0199 (15)	−0.0077 (11)	0.0016 (11)
O3	0.0268 (19)	0.059 (2)	0.0288 (17)	0.000	0.000	0.0176 (15)
O3'	0.0233 (17)	0.052 (2)	0.0278 (17)	0.000	0.000	0.0130 (12)
N1	0.0258 (16)	0.0330 (16)	0.0309 (14)	0.0067 (11)	0.0060 (11)	−0.0027 (10)
N1'	0.0240 (16)	0.0295 (16)	0.0303 (15)	0.0048 (12)	0.0025 (11)	0.0001 (11)
C1	0.046 (2)	0.030 (2)	0.062 (3)	0.0113 (16)	0.0114 (19)	0.0031 (16)
C1'	0.0327 (18)	0.0295 (18)	0.045 (2)	0.0088 (14)	0.0022 (14)	0.0057 (14)
C2	0.059 (4)	0.030 (3)	0.059 (4)	0.000	0.000	−0.010 (2)
C2'	0.047 (3)	0.024 (3)	0.050 (3)	0.000	0.000	−0.007 (2)
C3	0.0199 (13)	0.0347 (15)	0.0270 (13)	−0.0054 (9)	−0.0014 (15)	0.0028 (16)
C3'	0.0155 (14)	0.0348 (16)	0.0289 (14)	−0.0039 (11)	−0.0022 (12)	−0.0027 (12)
C4	0.0219 (16)	0.0346 (17)	0.0260 (15)	−0.0089 (12)	0.0026 (11)	−0.0019 (11)
C4'	0.0194 (15)	0.0294 (16)	0.0296 (15)	−0.0046 (12)	−0.0011 (11)	−0.0036 (11)
O4	0.063 (2)	0.099 (3)	0.074 (2)	−0.004 (2)	0.0086 (16)	0.037 (2)

### Geometric parameters (Å, °)

**Table:**

Cu1—N1^i^	2.019 (3)	N1'—H1'B	0.90
Cu1—N1	2.019 (3)	C1—C2	1.502 (6)
Cu1—O1	2.024 (2)	C1—H1C	0.97
Cu1—O1^i^	2.024 (2)	C1—H1D	0.97
Cu1—O3	2.180 (3)	C1'—C2'	1.513 (5)
Cu2—N1'^ii^	2.010 (3)	C1'—H1'C	0.97
Cu2—N1'	2.010 (3)	C1'—H1'D	0.97
Cu2—O1'	2.015 (3)	C2—C1^i^	1.502 (6)
Cu2—O1'^ii^	2.015 (3)	C2—H2A	0.97
Cu2—O3'	2.270 (3)	C2—H2B	0.97
O1—C3	1.259 (4)	C2'—C1'^ii^	1.513 (5)
O1'—C3'	1.262 (4)	C2'—H2'A	0.97
O2—C3	1.249 (5)	C2'—H2'B	0.97
O2'—C3'	1.247 (4)	C3—C4	1.502 (4)
O3—H3	0.85	C3'—C4'	1.507 (4)
O3'—H3'	0.85	C4—C4'	1.331 (4)
N1—C1	1.479 (5)	C4—H4	0.93
N1—H1A	0.90	C4'—H4'	0.93
N1—H1B	0.90	O4—H4A	0.86
N1'—C1'	1.475 (5)	O4—H4B	0.86
N1'—H1'A	0.90		
			
N1^i^—Cu1—N1	90.42 (18)	N1—C1—H1C	109.3
N1^i^—Cu1—O1	173.62 (11)	C2—C1—H1C	109.3
N1—Cu1—O1	88.93 (11)	N1—C1—H1D	109.3
N1^i^—Cu1—O1^i^	88.93 (11)	C2—C1—H1D	109.3
N1—Cu1—O1^i^	173.62 (11)	H1C—C1—H1D	107.9
O1—Cu1—O1^i^	91.00 (14)	N1'—C1'—C2'	111.3 (3)
N1^i^—Cu1—O3	97.70 (12)	N1'—C1'—H1'C	109.4
N1—Cu1—O3	97.70 (11)	C2'—C1'—H1'C	109.4
O1—Cu1—O3	88.68 (10)	N1'—C1'—H1'D	109.4
O1^i^—Cu1—O3	88.68 (10)	C2'—C1'—H1'D	109.4
N1'^ii^—Cu2—N1'	89.83 (19)	H1'C—C1'—H1'D	108.0
N1'^ii^—Cu2—O1'	175.68 (13)	C1—C2—C1^i^	113.6 (5)
N1'—Cu2—O1'	89.47 (10)	C1—C2—H2A	108.9
N1'^ii^—Cu2—O1'^ii^	89.47 (10)	C1^i^—C2—H2A	108.9
N1'—Cu2—O1'^ii^	175.68 (13)	C1—C2—H2B	108.9
O1'—Cu2—O1'^ii^	90.90 (15)	C1^i^—C2—H2B	108.9
N1'^ii^—Cu2—O3'	95.62 (11)	H2A—C2—H2B	107.7
N1'—Cu2—O3'	95.62 (11)	C1'—C2'—C1'^ii^	114.4 (5)
O1'—Cu2—O3'	88.69 (10)	C1'—C2'—H2'A	108.7
O1'^ii^—Cu2—O3'	88.69 (10)	C1'^ii^—C2'—H2'A	108.7
C3—O1—Cu1	124.7 (2)	C1'—C2'—H2'B	108.7
C3'—O1'—Cu2	126.3 (2)	C1'^ii^—C2'—H2'B	108.7
Cu1—O3—H3	109.6	H2'A—C2'—H2'B	107.6
Cu2—O3'—H3'	109.5	O2—C3—O1	125.1 (3)
C1—N1—Cu1	118.3 (2)	O2—C3—C4	116.3 (3)
C1—N1—H1A	107.9	O1—C3—C4	118.6 (3)
Cu1—N1—H1A	107.7	O2'—C3'—O1'	126.4 (3)
C1—N1—H1B	107.7	O2'—C3'—C4'	115.9 (3)
Cu1—N1—H1B	107.7	O1'—C3'—C4'	117.7 (3)
H1A—N1—H1B	107.1	C4'—C4—C3	123.2 (3)
C1'—N1'—Cu2	118.1 (2)	C4'—C4—H4	118.4
C1'—N1'—H1'A	107.9	C3—C4—H4	118.4
Cu2—N1'—H1'A	107.7	C4—C4'—C3'	123.3 (3)
C1'—N1'—H1'B	107.8	C4—C4'—H4'	118.4
Cu2—N1'—H1'B	107.6	C3'—C4'—H4'	118.4
H1'A—N1'—H1'B	107.2	H4A—O4—H4B	101.0
N1—C1—C2	111.8 (4)		
			
N1—Cu1—O1—C3	−55.8 (3)	Cu2—N1'—C1'—C2'	60.7 (4)
O1^i^—Cu1—O1—C3	117.8 (2)	N1—C1—C2—C1^i^	67.9 (6)
O3—Cu1—O1—C3	−153.5 (3)	N1'—C1'—C2'—C1'^ii^	−66.0 (6)
N1'—Cu2—O1'—C3'	63.3 (3)	Cu1—O1—C3—O2	−9.0 (5)
O1'^ii^—Cu2—O1'—C3'	−112.3 (3)	Cu1—O1—C3—C4	169.0 (2)
O3'—Cu2—O1'—C3'	159.0 (3)	Cu2—O1'—C3'—O2'	9.9 (5)
N1^i^—Cu1—N1—C1	42.8 (3)	Cu2—O1'—C3'—C4'	−169.8 (2)
O1—Cu1—N1—C1	−143.6 (3)	O2—C3—C4—C4'	137.3 (3)
O3—Cu1—N1—C1	−55.1 (3)	O1—C3—C4—C4'	−40.9 (4)
N1'^ii^—Cu2—N1'—C1'	−46.1 (3)	C3—C4—C4'—C3'	178.8 (3)
O1'—Cu2—N1'—C1'	138.2 (2)	O2'—C3'—C4'—C4	−142.2 (3)
O3'—Cu2—N1'—C1'	49.5 (3)	O1'—C3'—C4'—C4	37.5 (5)
Cu1—N1—C1—C2	−59.6 (4)		

Symmetry codes: (i) −*x*+1, *y*, *z*; (ii) −*x*, *y*, *z*.

### Hydrogen-bond geometry (Å, °)

**Table:**

*D*—H···*A*	*D*—H	H···*A*	*D*···*A*	*D*—H···*A*
O4—H4A···O2'	0.87	2.17	2.887 (5)	140
O3—H3···O2^iii^	0.85	1.88	2.713 (3)	166
O3'—H3'···O2'^iv^	0.85	1.91	2.708 (3)	156
N1—H1A···O1^v^	0.90	2.20	3.083 (4)	167
N1'—H1'A···O4^vi^	0.90	2.25	3.071 (5)	151
N1'—H1'B···O1'^vii^	0.90	2.26	3.135 (3)	164
O4—H4B···O2^viii^	0.86	1.99	2.785 (4)	153

Symmetry codes: (iii) −*x*+1, −*y*+1, *z*−1/2; (iv) −*x*, −*y*, *z*+1/2; (v) *x*, −*y*+1, *z*+1/2; (vi) *x*, −*y*, *z*+1/2; (vii) *x*, −*y*, *z*−1/2; (viii) *x*, *y*, *z*−1.
